# Supplementary material for: Effects of Chokeberries (Aronia spp.) on Cytoprotective and Cardiometabolic Markers and Semen Quality in 109 Mildly Hypercholesterolemic Danish Men: A Prospective, Double-Blinded, Randomized, Crossover Trial
Source: J Clin Med. 2023 Jan 3;12(1):373. doi: 10.3390/jcm12010373 (PMC9821700; doi:10.3390/jcm12010373)
Supplement: Supplementary file 1 [file jcm-12-00373-s001.zip › jcm-2076897-supplementary.pdf]

Supplementary Materials

Supplementary results from the paper: "The health effects of chokeberries (*Aronia* spp.) on a wide range of health markers in 109 mildly hypercholesterolemic Danish men: a prospective, double-blinded, randomized, crossover trial"

Table S1: Cross-over analysis on the effects of placebo (90 days) and Aronia powder administration (90 days) on blood levels of SOD, CAT, Isoprostanes , Haemoglobin A1c and . High Sensitive C-reactive Protein. The placebo and Aronia periods are separated by a 90 day washout period. Means represent follow-up measurements. (CI = Coefficient of variation).

			**Treatment period**		
**Variable**	**Treatment order**	**n**	**1**<br>Means (CI)	**2**<br>Means (CI)	**Difference Aronia-Placebo**<br>Means (CI)
**SOD U/ml**	Aronia then Placebo	51	6.8 (6.1;7.6)	5.5 (4.5;6.5)	1.3 (0.2;2.5)
	Placebo then Aronia	44	6.9 (6.0;7.8)	4.6 (3.8;5.5)	-2.3 (-3.7;-0.9)
	Treatment effect				-0.5 (-1.4;0.4)
**CAT, mU/ml**	Aronia then Placebo	51	1.6 (1.6;1.7)	1.7 (1.6;1.7)	-0.0 (-0.1;0.0)
	Placebo then Aronia	44	1.7 (1.6;1.7)	1.7 (1.6;1.7)	-0.0 (-0.1;0.1)
	Treatment effect				-0.0 (-0.1;0.0)
**Isoprostan, pg/mL**	Aronia then Placebo	50	957.5 (713.8;1201.1)	848.7 (636.6;1060.8)	108.8 (-116.8;334.4)
	Placebo then Aronia	44	1173.1 (618.7;1727.6)	970.9 (356.2;1585.7)	-202.2 (-1052.6;648.2)
	Treatment effect				-46.7 (-485.1;391.8)
**Haemoglobin A1c**	Aronia then Placebo	51	34.6 (33.6;35.6)	34.6 (33.7;35.6)	-0.0 (-0.4;0.4)
	Placebo then Aronia	44	35.8 (34.5;37.0)	35.6 (34.5;36.8)	-0.1 (-0.6;0.3)
	Treatment effect				-0.1 (-0.4;0.2)
**High Sensitive C-reactive Protein**	Aronia then Placebo	51	2.3 (1.2;3.3)	2.4 (1.6;3.1)	-0.1 (-1.2;1.0)
	Placebo then Aronia	44	1.8 (1.3;2.3)	3.0 (0.7;5.4)	1.3 (-0.9;3.5)
	Treatment effect				0.6 (-0.6;1.8)

Table S2: Cross-over analysis on the effects placebo (90 days) and Aronia powder administration (90 days) on a range of sperm quality parameters. The placebo and Aronia periods are separated by a 90 day washout period. Means represent follow-up measurements. (CI = Coefficient of variation).

			Treatment period		
**Variable**	**Treatment order**	**n**	**1**<br>Means (CI)	**2**<br>Means (CI)	**Difference Aronia-Placebo**<br>Means (CI)
Concentration of motile sperm (CMS) (/ml)	Aronia then Placebo	33	49.5 (35.6;63.3)	48.3 (34.1;62.5)	1.2 (-6.1;8.5)
	Placebo then Aronia	29	51.4 (32.2;70.5)	54.3 (32.6;75.9)	2.9 (-9.8;15.6)
	Treatment effect				2.0 (-5.2;9.2)
Total motile sperm count (TMSC)	Aronia then Placebo	33	155.4 (92.9;218.0)	145.6 (97.1;194.2)	9.8 (-27.3;46.9)
	Placebo then Aronia	29	154.0 (92.8;215.3)	166.9 (86.6;247.2)	12.9 (-37.4;63.2)
	Treatment effect				11.3 (-19.3;42.0)
Concentration of progressive motile (CPMS) (/ml)	Aronia then Placebo	33	32.8 (23.7;41.9)	30.7 (21.8;39.6)	2.1 (-3.4;7.6)
	Placebo then Aronia	29	36.3 (22.4;50.3)	41.0 (23.4;58.6)	4.7 (-5.5;14.9)
	Treatment effect				3.4 (-2.3;9.1)
Total progressive motile sperm count (TPMSC)	Aronia then Placebo	33	101.4 (64.3;138.6)	92.7 (62.2;123.2)	8.8 (-11.7;29.2)
	Placebo then Aronia	29	109.0 (64.3;153.8)	128.5 (58.3;198.7)	19.4 (-31.0;69.9)
	Treatment effect				14.1 (-12.8;41.0)
Sperm volume (ml)	Aronia then Placebo	33	3.0 (2.6;3.5)	3.0 (2.6;3.3)	0.1 (-0.2;0.3)
	Placebo then Aronia	29	3.1 (2.6;3.6)	2.9 (2.4;3.4)	-0.2 (-0.6;0.2)
	Treatment effect				-0.1 (-0.3;0.2)

Table S3: Cross-over analysis on the effects of placebo (90 days) and Aronia powder administration (90 days) on levels of blood glucose, HOMA and systolic/diastolic blood pressure. The placebo and Aronia periods are separated by a 90 day washout period. Means represent follow-up measurements. (CI = Coefficient of variation).

			Treatment period		
**Variable**	**Treatment order**	**n**	**1** Means (CI)	**2** Means (CI)	**Difference Aronia-Placebo** Means (CI)
Glucose	Aronia then Placebo	51	5.8 (5.7;6.0)	5.8 (5.6;5.9)	0.1 (0.0;0.2)
	Placebo then Aronia	44	5.9 (5.6;6.1)	5.9 (5.6;6.2)	0.0 (-0.1;0.2)
	Treatment effect				0.0 (-0.0;0.1)
HOMA	Aronia then Placebo	51	1.9 (1.6;2.2)	1.9 (1.6;2.3)	-0.0 (-0.2;0.2)
	Placebo then Aronia	44	2.2 (1.5;2.9)	2.0 (1.4;2.5)	-0.2 (-0.7;0.2)
	Treatment effect				-0.1 (-0.4;0.1)
Blood pressure, systolic (mmHg), left arm	Aronia then Placebo	51	127.8 (123.7;132.0)	130.2 (126.5;134.0)	-2.4 (-4.7;-0.1)
	Placebo then Aronia	43	124.9 (120.9;129.0)	128.1 (123.8;132.4)	3.2 (0.9;5.4)
	Treatment effect				0.4 (-1.2;2.0)
Blood pressure, diastolic (mmHg), left arm	Aronia then Placebo	51	84.3 (81.6;87.0)	86.5 (83.8;89.1)	-2.2 (-3.5;-0.9)
	Placebo then Aronia	43	83.4 (80.0;86.8)	84.4 (81.0;87.8)	0.9 (-0.6;2.4)
	Treatment effect				-0.6 (-1.6;0.3)
Blood pressure, systolic (mmHg), right arm	Aronia then Placebo	50	129.3 (125.3;133.4)	130.0 (126.4;133.5)	-0.6 (-2.8;1.5)
	Placebo then Aronia	43	126.6 (122.5;130.7)	128.3 (124.4;132.3)	1.8 (-0.2;3.8)
	Treatment effect				0.6 (-0.9;2.0)
Blood pressure, diastolic (mmHg), right arm	Aronia then Placebo	50	85.5 (82.8;88.2)	86.6 (84.1;89.1)	-1.1 (-2.4;0.2)
	Placebo then Aronia	43	83.5 (80.4;86.6)	84.7 (81.5;87.8)	1.2 (-0.2;2.6)
	Treatment effect				0.0 (-0.9;1.0)
